# Acylsucrose-Producing Tomato Plants Forces *Bemisia tabaci* to Shift Its Preferred Settling and Feeding Site

**DOI:** 10.1371/journal.pone.0033064

**Published:** 2012-03-09

**Authors:** Maria Jose Rodríguez-López, Elisa Garzo, Jean Patrick Bonani, Rafael Fernández-Muñoz, Enrique Moriones, Alberto Fereres

**Affiliations:** 1 Instituto de Ciencias Agrarias (ICA), Consejo Superior de Investigaciones Científicas, Madrid, Spain; 2 Instituto de Hortofruticultura Subtropical y Mediterránea “La Mayora”, Consejo Superior de Investigaciones Científicas (IHSM-UMA-CSIC), Málaga, Spain; Max Planck Institute for Chemical Ecology, Germany

## Abstract

**Background:**

The whitefly *Bemisia tabaci* (Genn.) causes dramatic damage to plants by transmitting yield-limiting virus diseases. Previous studies proved that the tomato breeding line ABL 14-8 was resistant to *B. tabaci*, the vector of tomato yellow leaf curl disease (TYLCD). This resistance is based on the presence of type IV glandular trichomes and acylsucrose production. These trichomes deter settling and probing of *B. tabaci* in ABL 14-8, which reduces primary and secondary spread of TYLCD.

**Methodology/Principal Findings:**

Whitefly settlement preference was evaluated on the adaxial and abaxial leaf surfaces of nearly-isogenic tomato lines with and without *B. tabaci*-resistance traits, ‘ABL 14-8 and Moneymaker’ respectively, under non-choice and free-choice conditions. In addition, the Electrical Penetration Graph technique was used to study probing and feeding activities of *B. tabaci* on the adaxial and abaxial leaf surfaces of the same genotypes. *B. tabaci* preferred to settle on the abaxial than on the adaxial surface of ‘Moneymaker’ leaves, whereas no such preference was observed on ABL 14-8 tomato plants at the ten-leaf growth stage. Furthermore, *B. tabaci* preferred to feed on the abaxial than on the adaxial leaf surface of ‘Moneymarker’ susceptible tomato plants as shown by a higher number of sustained phloem feeding ingestion events and a shorter time to reach the phloem. However, *B. tabaci* standard probing and feeding behavior patterns were altered in ABL 14-8 plants and whiteflies were unable to feed from the phloem and spent more time in non-probing activities when exposed to the abaxial leaf surface.

**Conclusions/Significance:**

The distorted behavior of *B. tabaci* on ABL 14-8 protects tomato plants from the transmission of phloem-restricted viruses such as *Tomato yellow leaf curl virus* (TYLCV), and forces whiteflies to feed on the adaxial side of leaves where they feed less efficiently and become more vulnerable to natural enemies.

## Introduction

Plants use passive and active defense strategies to limit pathogen and pest damage [1]. Many plants display chemical deterrents on their cuticular surface or store toxic compounds in vacuoles or trichomes for release on tissue damage. These preformed structures and metabolites provide an immediate defense against the attacking insect by deterring colonization and the intrinsic rate of population increase. Glandular and non-glandular trichomes affect herbivore settling and survival on host plants [2]. Glandular trichomes produce array of volatile and non-volatile secondary metabolites including acylsugars, terpenoids, phenylpropanoids and flavonoids [2], [3]. In *Solanum* spp., glandular trichomes types IV and VI are associated with high levels of resistance to diverse arthropod species including aphids and whiteflies [4], [5]. Quantitative trait loci control glandular trichomes IV and VI mediated resistance, which is correlated with acylsugar or toxic phenolic compound production [5]–[7]. One system of pest resistance that is largely due to deterrence is the resistance in various species in the *Solanaceae* family based upon the production of acylsugars, which are secondary metabolites produced by type IV glandular trichomes. These acylsugars mediate the resistance to many tomato pests in the wild species *S. pennellii* [8]. Appropriate application of the pure acylsugars from *S. pennellii* LA716 reduces feeding of aphids *Myzus persicae* and *Macrosiphum euphorbiae* [9]–[11] and sharply reduces oviposition and feeding of the leafminer *Liriomyza trifolii* [12] and whitefly *Bemisia tabaci* [13]. Goffreda et al. [10] described that acylsugars are irritants and deter insect settling and phloem feeding and increase the frequency of probing.

Host plant resistance to insect vectors is one of the best strategies to manage circulative vector-borne virus diseases [14]. Plant virus transmission by insect vectors is entirely dependent on the behavior and dispersal capacity of their vectors to spread virus from plant to plant. Host plant selection is particularly important among insects such as whiteflies. As hemipteran vectors of plant viruses, whiteflies follow a series of steps to search and find their host plants and identify adequate settling and feeding sites. These steps are a series of successive events that culminate in sustained phloem sap ingestion once the host plant is recognized as an acceptable source for feeding [15]. Whitefly stylet activities into the phloem sieve elements are linked to the transmission of phloem-restricted persistent viruses, such as *Tomato yellow leaf curl virus* (TYLCV, genus *Begomovirus*, family *Geminiviridae*) [16].

Most hemipterans prefer to walk and settle on the abaxial side of the leaf soon after landing. For instance, aphids such as *M. persicae* prefer to settle on the abaxial surface of the leaf rather than in the adaxial surface [17]. Crawlers of *B. tabaci* also prefer to settle on the abaxial (80–100%) than on the adaxial (0–20%) surface of the leaf when given a choice [18]. After contact with the plant surface, *B. tabaci* evaluates host plant quality by labial dabbing and probing using its piercing mouthparts. Probing and feeding behavior by piercing sucking insects can be monitored very closely by means of electronic devices. A useful tool to study the probing behavior of whiteflies is the electronic monitoring system, which was developed by Mc Lean and Kinsey [19] using AC amplifiers and later modified by Tjallingii [20] using a DC system. This technique also called electrical penetration graph (EPG) [21] has been successfully used to understand the feeding behavior of the whiteflies *Trialeurodes vaporariorum* and *B. tabaci* [16], [22]–[26]. The technique consists of assembling an electric circuit that includes the insect and the plant. Stereotypical voltage fluctuations (waveforms) are associated with specific stylet activities, based on correlations with other techniques that indicate the precise stylet tip position or the occurrence of ingestion, egestion, or salivation events [27]. The EPG technique can be used to locate the specific tissues in which plant resistance factors are operating [28].

In a recent work, we studied the host preference and the probing behavior of *B. tabaci* as well as the spread of tomato yellow leaf curl disease (TYLCD) on the whitefly-resistant tomato line, ABL 14-8 and the whitefly-susceptible cv. Moneymaker [29]. ABL 14-8 is a breeding line in which the presence of type IV leaf glandular trichomes and secretion of acylsucroses were introgressed from the wild tomato *Solanum pimpinellifolium* L. accession TO-937 into the background of its nearly-isogenic line, cv. ‘Moneymaker’. Results of preference bioassays indicated that the presence of these traits may deter alighting and settling of *B. tabaci* on ABL 14-8. Moreover, EPG studies indicated that *B. tabaci* spent more time in non-probing activities and showed a reduced ability to start probing. Such behavior resulted in a reduced ability to reach the phloem. Interestingly, these resistance characters in tomato were shown to be effective to reduce significantly primary and secondary spread of TYLCD-causing *Tomato yellow leaf curl virus* (TYLCV) [29].

As type-IV glandular trichomes are mainly located on the abaxial leaf surface of the genotype carrying the whitefly-resistance traits, we hypothesized that *B. tabaci* would elude to settle and feed on the abaxial side of leaves. These changes in behavior may have important implications on the transmission of TYLCV and on the survivorship of *B. tabaci*. Therefore, the aim of the present work was to evaluate the influence of the adaxial and the abaxial leaf surface of the nearly-isogenic tomato lines with and without *B. tabaci*-resistance traits, ABL 14-8 and ‘Moneymaker’ respectively, on *B. tabaci* settling and probing behavior.

## Methods

### Tomato plants and whitefly population

The tomato lines used in this work were the *B. tabaci* and virus susceptible tomato cv. Moneymaker and its nearly-isogenic whitefly-resistant line ABL 14-8. The type IV glandular trichomes and acylsucrose secretion on the abaxial leaf surface of the ABL 14-8 line were introgressed by three recurrent backcrosses and selfing steps from the wild tomato *Solanum pimpinellifolium* L., accession TO-937 to the *S. lycopersicum* L. cultivar ‘Moneymaker’, which lacks those traits [30], [31]. The Estación Experimental “La Mayora” – CSIC germplasm collection accession TO-937 is an inbred line derived from *S. pimpinellifolium* material collected by our colleague J. Cuartero at 50 m altitude on the coastal plain of Lambayeque Department, Peru in 1983. The original accession segregated widely for density of glandular trichomes and it was fixed by four consecutive selfing and selection steps.

Plantlets of ABL 14-8 and ‘Moneymaker’ were individually sown in pots (18 cm diameter) containing plant-nutrient and vermiculite (1∶1 v/v) and grown in a glasshouse with a temperature of 25±3°C and a relative humidity of 75±5%. To conduct experiments, we had to consider the plant growth stage because the full expression of acylsucrose production in the *B. tabaci*-resistant-donor genotype TO-937 and in ABL 14-8 occurs only after the ten-leaf growth stage [29], [32]. Plants at four and ten-leaf growth stage were used in the experiments.

*B. tabaci* individuals were obtained from a Q biotype colony originated from individuals collected in Málaga (Spain) and reared on melon (*Cucumis melo* L. cv. ‘Primal’) plants within wooden cages covered with insect-proof nets in a growth chamber (25°C day and 20°C night, 70% RH with a photoperiod of 16∶8 h Light∶Dark and 250 µmol s^−1^ m^−2^ of photosynthetically active radiation).

### *B. tabaci* settling under non-choice and free-choice conditions

**Figure 1:**
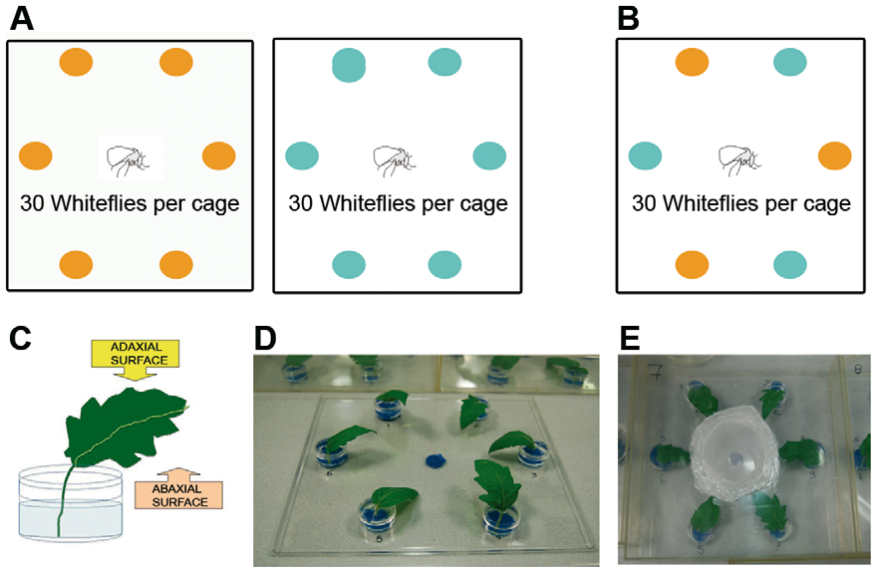
Preference test under non-choice and free-choice conditions. **A**. For non- choice conditions, six leaflets of each nearly-isogenic tomato line (‘Moneymaker’: orange; ABL 14-8: blue) were placed forming a circle in independent trays. **B**. For free-choice conditions, three leaflets of each line (six leaflets per tray) were placed in an alternate design. **C**. Petiole leaflets were inserted into a plastic dish (2 cm Ø×1 cm high) with nutritive solution to offer both adaxial and adaxial leaf surfaces to whiteflies. **D**. Six leaflets per tray were placed abaxial surface down with their tips directed to the center of the circle formed by the leaflets. **E**. The plastic tray was covered with a plastic lid (25×25×10 cm) with an opening covered with muslin for ventilation.

Settlement preference of adult individuals of the whitefly *B. tabaci* was evaluated on the adaxial and abaxial leaf surfaces of resistant ABL 14-8 and the *B. tabaci*-susceptible ‘Moneymaker’ tomato plants under non-choice and free-choice conditions in the ICA-CSIC facilities at Madrid, Spain. Detached leaflets from tomato plants of ‘Moneymaker’ and ABL 14-8 at four and ten-leaf growth stages were used. For non-choice experiments, six leaflets of each genotype and growth stage tested were placed forming a circle in independent plastic trays (25×25 cm) (Figure 1A). For free-choice experiments, three leaflets of each genotype (six leaflets per tray) were placed in an alternate design in a single plastic tray (Figure 1B). Each leaflet petiole was inserted in a plastic dish (2 cm diameter×1 cm high) filled with nutritive solution (0.25 g/l of Nutrichem 60, Miller Chemical, Hanover, PE, EE.UU.) to maintain leaflet turgor (Figure 1C). Leaflets were placed abaxial surface down and at an angle with the horizontal plane (so that both leaflet surfaces were freely accessible to whiteflies), with their tips directed to the center of the circle formed by the leaflets (Figure 1D). Thirty adult whiteflies (5 whiteflies per leaflet) without distinction of sex were released in the middle of the circle after a short cold treatment at 4°C during 10 min to reduce their activity and facilitate handling. Then, each plastic tray was covered with a plastic lid (25×25×10 cm) with an opening covered with muslin for ventilation (Figure 1E). The trays were then placed in a growth chamber (25°C constant temperature, 16∶8 h Light∶Dark) photoperiod, 65% RH). The number of whiteflies settled on both adaxial and abaxial leaflet surfaces were counted at 0.5, 1, 2, 4, 8, 24 and 48 h after release. Some whiteflies refused to settle on the leaflets and remained on the plastic tray or lid and were not considered for data analysis. Free-choice and non-choice experiments were replicated 12 times. Data, calculated as the mean number of whiteflies per six leaflets in non-choice conditions and number of whiteflies per three leaflets in free-choice conditions on each genotype and leaflet side (adaxial or abaxial) at different time intervals were subjected to Mann-Whitney U-tests at 0.05 significance level, using the SPSS v. 17 software (SPSS Inc., Chicago, IL, USA).

### Electrical Penetration Graphs of *B. tabaci* on adaxial and abaxial side of ‘Moneymaker’ and ABL 14-8 leaflets

The DC-EPG technique was used in the ICA-CSIC facilities to monitor probing and feeding activities of female *B. tabaci* adults on ‘Moneymaker’ and ABL 14-8 plants during 6-h periods. Before EPG recordings began, the whiteflies were subjected to a short cold treatment to facilitate handling as described above. Then, an extra thin gold wire (2-cm length, 12.5-µm diameter) was attached to the dorsum of the whitefly with a small droplet of water based silver glue paint. The opposite extreme of the gold wire was attached to a copper electrode. Then, whiteflies were starved for 30 min for management recovery. The copper electrode was connected to the input of the first stage amplifier with a 1 GΩ input resistance and 50× gain [33], [34] and whiteflies were placed on either the adaxial or the abaxial leaf surface of the tomato test plants at the ten-leaf growth stage. The EPG device, computer software and environmental conditions were the same as the ones described in a previous study (29). Each whitefly was used only once and each plant was used a maximum of three times for EPG recording. Whitefly probing-associated EPG waveforms were recorded and analyzed: waveform np, non-probing behavior (no stylet contact with the leaf tissue); waveform C, intercellular apoplastic stylet pathway where the insects show a cyclic activity of mechanical stylet penetration and secretion of saliva; waveform pd (potential drops), represents brief (4–12 sec) intracellular stylet punctures during the pathway phase (C). Also, two waveforms related with the phloem activity were recorded: waveform E1, salivation into phloem sieve elements at the beginning of the phloem phase [16]; and waveform E2, correlated with passive phloem sap uptake from the sieve elements that is comparable to E2 of aphids [35]. EPG sequential and non sequential parameters related to the pathway (C and pd), and phloem phase (E1 and E2) were calculated for each of the EPG recordings using the MS Excel workbook for automatic parameter calculation of EPG data (version 4.0) developed by Sarria et al. [36]. Data from 13–16 individual adult female whiteflies were recorded for each tomato genotype and leaf surface. Selected EPG parameters (mean ± standard error) were calculated and compared between treatments as described in Backus et al. [37]: PPW, proportion of individuals that produced the waveform type; NWEI, number of waveform events per insect; WDI, waveform duration (min) per insect; and WDE, waveform duration (min) per event. Data were subjected to Mann-Whitney U-tests at a 0.05 significance level, using the SPSS v. 17 software.

## Results

### Effects of tomato leaf surface on *B. tabaci* preference under non-choice and free-choice conditions

**Figure 2:**
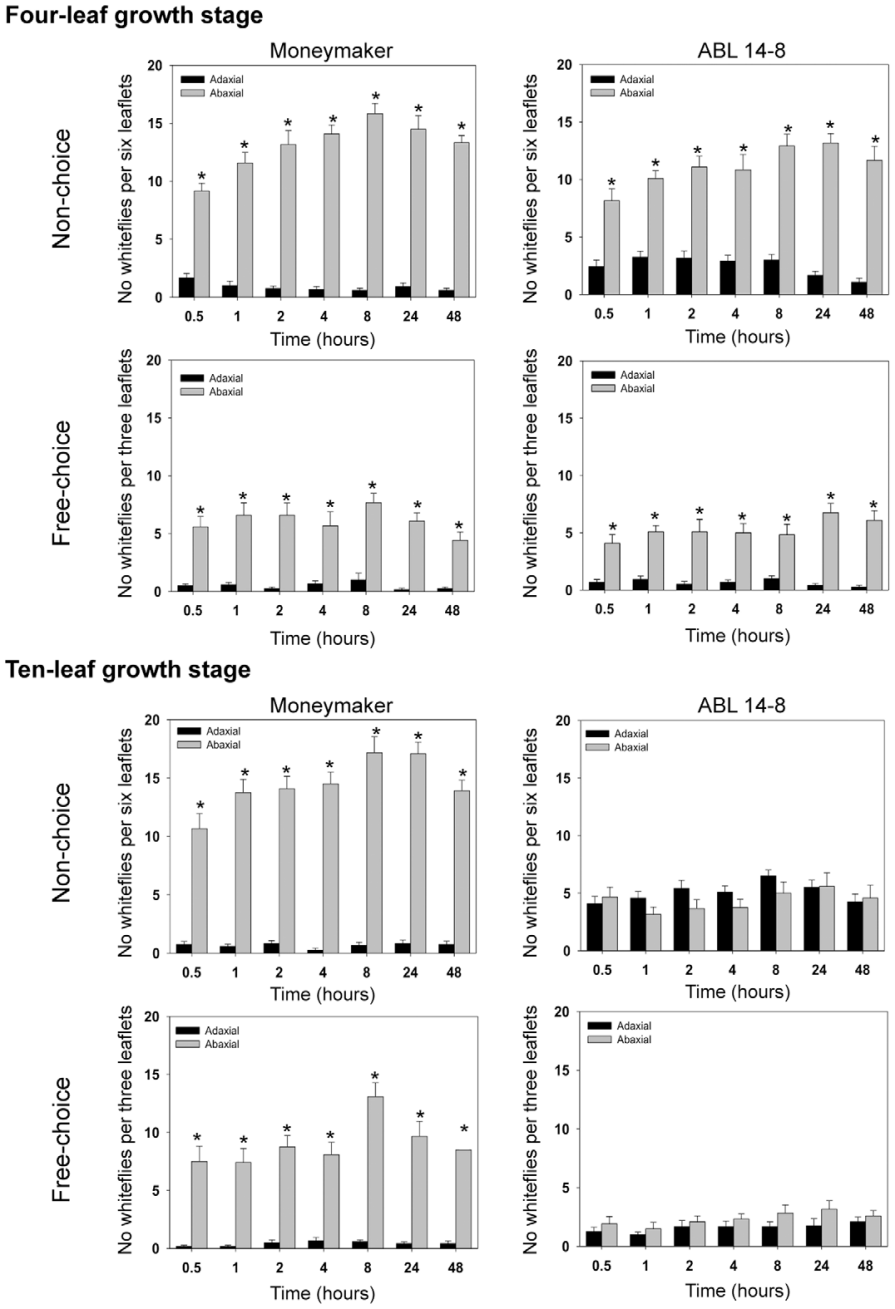
Whiteflies settling on the adaxial and abaxial leaflet surfaces of ‘Moneymarker’ and ABL 14-8. Free-choice and non-choice preference assays of *B. tabaci* on the adaxial and abaxial leaflet surfaces of‘Moneymaker’ and ABL 14-8 nearly-isogenic tomato lines at four and ten-leaf growth stage. Mean number of whiteflies per six leaflets under non-choice conditions and mean number of whiteflies per three leaflets under free-choice conditions on the adaxial (black bars) and abaxial (grey bars) leaflet surfaces of ‘Moneymaker’ and ABL 14-8 at different time intervals after release of 30 whiteflies per test in the centre of the arena. Asterisks indicate significant differences (P<0.05) between both the abaxial and the adaxial leaf surfaces at each time interval using Mann-Whitney U-test. Bars indicate the standard error of the mean.

Whiteflies did not reject the abaxial surface of ABL 14-8 leaves at the four-leaf growth stage probably due to the absence of acylsucrose in the glandular type IV-trichomes. Acylsucrose production is fully expressed in glandular trichomes only after the ten-leaf growth stage [32]. We also observed a significantly (P<0.05) higher number of whiteflies on the abaxial than on the adaxial leaf surface on both ABL 14-8 and ‘Moneymaker’ under free-choice and non-choice conditions at the four-leaf growth stage (Figure 2).

At the ten-leaf growth stage (Figure 2), a significantly higher number of whiteflies was also counted on the abaxial than on the adaxial side of ‘Moneymaker’ leaflets since early times after release either for free-choice and for non-choice conditions. However, no significant differences were observed in the number of whiteflies settling on the abaxial and on the adaxial ABL 14-8 leaf surfaces under both non-choice and free choice conditions. Therefore, the abaxial side of leaves which is the ideal settling site was not preferred by *B. tabaci* when exposed to ABL 14-8 leaflets. No significant number of dead whiteflies was observed in any treatment.

### Probing and feeding behavior of *B. tabaci* on adaxial and abaxial tomato leaf surfaces

We evaluated the probing and feeding behavior of *B. tabaci* in both leaf surfaces of ‘Moneymaker’ and ABL 14-8, at the ten-leaf growth stage, when acylsucrose production is fully expressed. When the adaxial and abaxial leaf surfaces of ‘Moneymaker’ plants were compared (Table 1), *B. tabaci* clearly preferred to feed on the abaxial leaf surface as shown by a significantly higher number of sustained feeding ingestion (E2>10 min) events from the phloem sieve elements (abaxial: 0.5±0.2 *vs.* adaxial: 0.1±0.1) and shorter time to reach the phloem (Table 1). The data also revealed a significantly lower duration (min) of C waveform (intercellular stylet pathway) per insect (Waveform Duration per Insect) (P<0.05) on the abaxial leaf surface (111.1±13.8) compared with the adaxial surface (154.6±14.4). The number of probes to reach the phloem (Number of probes to the 1st E1) was significantly lower (P<0.05) on the abaxial (22.8±5.8) than on the adaxial (55.3±12.1) leaf surface. These results suggest that *B. tabaci* reached the phloem much easier when approaching the abaxial than the adaxial side of the leaves of susceptible ‘Moneymaker’ plants. However, standard probing and feeding behavior patterns of *B. tabaci* were altered in the ABL 14-8 plants (Table 2), in which type IV glandular trichomes and acylsucrose accumulation are present on the abaxial surface of leaves. In contrast to the results obtained on the susceptible genotype, a significant (P<0.005) longer duration (min) of non-probing activity of whiteflies was obtained on the abaxial (272.2±14.8) than on the adaxial (206.2±18.2) leaf surface of ABL 14-8 plants. The mean duration (min) of the probing activity per insect (Waveform Duration per Insect), moreover, was lower on the abaxial (86.3±15.1) than on the adaxial (149.4±19.0) leaf surface of the resistant genotype. Therefore, *B. tabaci* spent much more time on non-probing activities on the abaxial than on the adaxial leaf surface of ABL 14-8 tomato plants. Furthermore, *B. tabaci* was unable to feed from the phloem sieve elements when exposed to the abaxial surface of ABL 14-8 leaves, as no E2 events were recorded. No significant differences were observed between adaxial and abaxial leaf surfaces for phloem-related variables in the whitefly-resistant genotype, which suggests that there is no resistance at the phloem level.

**Table 1:** Mean (± standard error) sequential and non-sequential EPG variable values (ranges in parenthesis) for *B. tabaci* probing behavior on the abaxial and adaxial leaf surface of ten-leaf growth stage ‘Moneymaker’ plants during a six-hour recording^a^.

Non-sequential variables	Surface	PPW	NWEI	P^b^	WDI	P	WDE	P
Non-probe	Adaxial	15/15	51.3±6.3 (13–100)	0.16	192.8±15.4 (94.1–275.8)	0.61	5.4±1.3 (1.2–21.2)	0.64
	Abaxial	16/16	43.9±8.6 (13–128)		200.3±20.6 (56.6–291.8)		8.0±1.9 (1.5–22.0)	
Probe	Adaxial	15/15	50.7±6.3 (12–99)	0.17	165.9±15.6 (79.7–265.7)	0.61	3.9±0.6 (1.7–8.6)	0.13
	Abaxial	16/16	43.3±8.6 (12–128)		158.0±20.7 (68.0–302.4)		4.9±0.7 (0.9–10.4)	
C	Adaxial	15/15	51.3±6.5 (12–100)	0.25	154.6±14.4 (62.6–261.8)	0.04	3.7±0.6 (1.5–8.6)	0.58
	Abaxial	16/16	44.8±8.5 (13–128)		111.1±13.8 (34.2–187.7)		3.1±0.4 (0.9–5.6)	
pd^c^	Adaxial	9/15	3.9±1.9 (0–25)	0.08	38.6±22.9 (2.5–213.7)	0.07	4.3±0.7 (1.9–8.5)	0.94
	Abaxial	12/16	8.0±2.1 (0–25)		40.3±7.6 (6.6–91.9)		4.3±0.6 (2.3–9.1)	
E1	Adaxial	3/15	0.5±0.3 (0–3)	0.04	4.3±1.8 (1.5–7.6)	0.86	1.8±0.4 (1.3–2.5)	0.87
	Abaxial	10/16	1.1±0.3 (0–4)		8.0±3.9 (0.4–41.6)		4.0±1.9 (0.4–20.8)	
E2	Adaxial	2/15	0.1±0.1 (0–1)	0.02	31.2±29.0 (2.1–60.2)	0.60	31.2±29.0 (2.1–60.2)	0.79
	Abaxial	8/16	0.8±0.3 (0–4)		67.2±20.9 (0.7–142.8)		40.8±14.6 (0.7–130.6)	
Sequential variables								
Time to 1st probe from start of EPG	Adaxial	15/15			50.8±19.9 (0.1–224.6)	0.11		
	Abaxial	16/16			5.5±2.5 (0.1–39.8)			
Duration of 1st probe	Adaxial	15/15			2.8±0.8 (0.1–10.3)	0.64		
	Abaxial	16/16			3.6±2.1 (0.1–34.5)			
Duration of 2nd probe	Adaxial	15/15			1.4±0.3 (0.1–5.1)	0.34		
	Abaxial	16/16			4.0±3.1 (0.2–50.4)			
Number of brief probes(<1 min)	Adaxial	15/15	28.3±4.9 (3–75)	0.49				
	Abaxial	16/16	29.2±7.7 (3–116)					
Time from the beginning of that probe to 1st E	Adaxial	3/15			295.8±33.6 (19.1–360.0)	0.00		
	Abaxial	10/16			146.0±42.5 (4.5–359.9)			
Time from 1st probe to 1st E	Adaxial	3/15			334.5±14.2 (171.5–360.0)	0.00		
	Abaxial	10/16			217.6±33.1 (13.7–359.9)			
Number of probes to the 1st E1	Adaxial	3/15	55.3±12.1 (39–79)	0.03				
	Abaxial	10/16	22.8±5.8 (5–70)					
Number of probes after 1st E	Adaxial	3/15	2.8±2.2 (0–33)	0.04				
	Abaxial	10/16	8.3±3.4 (0–49)					
Number of probes (shorter than 3 min) after 1st E	Adaxial	3/15	1.7±1.3 (0–19)	0.04				
	Abaxial	10/16	7.2±3.1 (0–46)					
Total duration of E1 followed by E2	Adaxial	2/15			1.6±0.1 (1.5–1.7)	0.30		
	Abaxial	8/16			8.6±4.1 (0.4–36.3)			
Number of sustained E2 (longer than 10 min)	Adaxial	1/15	0.1±0.1 (0–1)	0.04				
	Abaxial	6/16	0.5±0.2 (0–3)					
Total duration of E1 followed by sustained E2 (>10 min)	Adaxial	1/15			1.5±0.0 (1.5–1.5)	0.62		
	Abaxial	6/16			9.3±5.6 (0.5–36.3)			

a PPW, proportion of individuals that produced a given waveform type; NWEI, number of waveform events per insect; WDI, waveform duration (min) per insect; WDE, waveform duration (min) per event. Non-probe, non-probing activity, no stylet contact with the plant tissue; probe, stylet insertion into the plant of any duration or purpose; waveform C, intercellular apoplastic stylet pathway where the insects show a cyclic activity of mechanical stylet penetration and secretion of saliva; waveform pd (potential drop), represents brief (4 to 12 s) intracellular stylet punctures during the pathway phase (C). There are two waveforms related with the phloem activity: waveform E1, salivation into the phloem sieve elements at the beginning of the phloem phase [16]; waveform E2, correlated with passive phloem sap uptake from the sieve elements that is comparable to E2 of aphids [35].

b Statistical comparisons between the two leaflets surfaces for each parameter were made by using the nonparametric Mann Whitney U test.

c Potential drop (pd) duration is expressed in seconds.

**Table 2:** Mean (± standard error) sequential and non-sequential EPG variable values (ranges in parenthesis) for *B. tabaci* probing behavior on abaxial and adaxial leaf surface at ten-leaf growth stage ABL 14-8 plants during a six-hour recording^a^.

Non-sequential variables	Surface	PPW	NWEI	P^b^	WDI	P	WDE	P
Non-probe	Adaxial	15/15	49.8±9.0 (9–131)	0.10	206.2±18.2 (74.8–284.9)	0.01	7.8±2.0 (1.2–29.9)	0.02
	Abaxial	13/13	28.6±5.3 (5–68)		272.2±14.8 (178.9–336.3)		17.1±4.5 (3.7–60.7)	
Probe	Adaxial	15/15	49.4±9.0 (9–131)	0.11	149.4±19.0 (66.7–285.0)	0.01	5.2±1.3 (0.9–14.5)	0.80
	Abaxial	13/13	28.0±5.3 (4–67)		86.3±15.1 (23.6–180.8)		4.9±1.5 (0.8–20.1)	
C	Adaxial	15/15	50.3±9.0 (10–131)	0.10	112.9±18.6 (13.5–219.7)	0.20	3.6±1.1 (0.2–14.5)	0.08
	Abaxial	13/13	28.3±5.3 (4–67)		74.8±12.7 (23.6–154.2)		3.7±0.8 (0.8–12.8)	
pd^c^	Adaxial	9/15	4.1±1.4 (0–18)	0.38	25.3±7.0 (7.2–61.3)	0.49	3.7±0.2 (3.0–4.4)	0.87
	Abaxial	13/13	1.7±0.5 (0–6)		14.2±3.5 (2–32.1)		5.2±1.9 (2–16)	
E1	Adaxial	3/15	0.3±0.2 (0–2)	0.70	8.0±7.0 (0.9–22.0)	0.56	7.8±7.1 (0.5–22.0)	0.56
	Abaxial	2/13	0.2±0.1 (0–1)		5.7±3.8 (1.8–9.5)		5.7±3.8 (1.8–9.5)	
E2	Adaxial	3/15	0.3±0.2 (0–2)	0.08	39.8±25.3 (2.8–88.2)	-	35.1±26.8 (2.8–88.2)	-
	Abaxial	0/13	0.0±0.0 (0-0)		-		-	
Sequential variables								
Time to 1st probe from start of EPG	Adaxial	15/15			15.1±6.4 (0.2–92.8)	0.08		
	Abaxial	13/13			72.5±23.3 (0.3–263.6)			
Duration of 1st probe	Adaxial	15/15			2.6±1.8 (0.1–27.9)	0.26		
	Abaxial	13/13			1.5±0.4 (0.1–5.3)			
Duration of 2nd probe	Adaxial	15/15			3.7±2.1 (0.1–30.5)	0.84		
	Abaxial	13/13			10.3±9.4 (0.3–123.4)			
Number of brief probes(<1 min)	Adaxial	15/15	27.1±6.1 (5–96)	0.34				
	Abaxial	13/13	18.5±3.9 (2–45)					
Time from the beginning of that probe to 1st E	Adaxial	3/15			294.5±34.8 (8.6–359.9)	0.76		
	Abaxial	2/13			306.6±35.1 (7.4–359.9)			
Time from 1st probe to 1st E	Adaxial	3/15			316.9±24.4 (89.6–359.9)	0.86		
	Abaxial	2/13			338.1±13.8 (208.3–359.9)			
Number of probes to the 1st E1	Adaxial	3/15	34.7±9.1 (17–47)	0.56				
	Abaxial	2/13	31.0±6.0 (25–37)					
Number of probes after 1st E	Adaxial	3/15	7.1±5.1 (0–69)	1.00				
	Abaxial	2/13	2.2±1.5 (0–16)					
Number of probes (shorter than 3 min) after 1st E	Adaxial	3/15	5.7±4.2 (0–60)	1.00				
	Abaxial	2/13	2.1±1.4 (0–15)					
Total duration of E1 followed by E2	Adaxial	3/15			8.0±7.0 (0.9–22.0)	-		
	Abaxial	0/13			-			
Number of sustained E2 (longer than 10 min)	Adaxial	2/15	0.2±0.1 (0–2)	0.18				
	Abaxial	0/13	0.0±0.0 (0-0)					
Total duration of E1 followed by sustained E2(>10 min)	Adaxial	2/15			11.6±10.5 (1.1–22.0)	-		
	Abaxial	0/13			-			

a PPW, proportion of individuals that produced a given waveform type; NWEI, number of waveform events per insect; WDI, waveform duration (min) per insect; WDE, waveform duration (min) per event. Non-probe, non-probing activity, no stylet contact with the plant tissue; probe, stylet insertion into the plant of any duration or purpose; waveform C, intercellular apoplastic stylet pathway where the insects show a cyclic activity of mechanical stylet penetration and secretion of saliva; waveform pd (potential drop), represents brief (4 to 12 s) intracellular stylet punctures during the pathway phase (C). There are two waveforms related with the phloem activity: waveform E1, salivation into the phloem sieve elements at the beginning of the phloem phase [16]; waveform E2, correlated with passive phloem sap uptake from the sieve elements that is comparable to E2 of aphids [35].

b Statistical comparisons between the two leaflets surfaces for each parameter were made by using the nonparametric Mann Whitney U test.

c Potential drop (pd) duration is expressed in seconds.

## Discussion

It has been repeatedly reported that *B. tabaci* as well as other pierce-sucking insects prefer to settle, feed and oviposit on the abaxial than on the adaxial leaf surface of their host plants [4], [17]. Our assays for settlement preference under free-choice conditions on leaflets of ten-leaf growth stage plants also showed that *B. tabaci* clearly preferred to settle on the abaxial surface of leaflets of the susceptible ‘Moneymaker’ cultivar. However, whiteflies showed no settling preference on ABL 14-8 plants. Furthermore, a much higher mean number of whiteflies per leaflet was observed on the adaxial surface of ABL 14-8 than on ‘Moneymaker’ (Figure 2). These observations demonstrated that the abaxial surface of ABL 14-8 leaves was not the preferred settling site for whiteflies in contrast to what was observed in leaves of the *B. tabaci*-susceptible ‘Moneymaker’ plants. This same result is accentuated under non-choice conditions, where whiteflies clearly preferred to settle on the abaxial surface of ‘Moneymaker’ but not on the abaxial side of ABL 14-8 leaflets. Therefore, our results on settlement preference clearly showed that the ABL 14-8 genotype modifies the innate behavior of *B. tabaci* for settling and feeding on the abaxial surface of tomato leaves. This altered behavior is likely due to the presence of deterrent acylsugars secreted by the type IV glandular trichomes, which are mainly located on the abaxial surface of the whitefly-resistant genotype ABL 14-8 leaves. Actually, we found out that type IV glandular trichomes were very abundant on the abaxial leaf surface (10.20±1.11 units/mm^2^) of ABL 14-8 plants at the ten-leaf growth stage. Such trichomes were almost absent on the adaxial leaf surface (0.07±0.03 units/mm^2^). Furthermore, it has been shown that acylsucrose is an antixenotic factor that deters whitefly settling and delays stylet penetration by whiteflies on ABL 14-8 leaves [29]. A similar effect of the leaf surface and the type of foliar trichome (glandular and non glandular) on *B. tabaci* was observed in tomato by Oriani and Vendramim [4]. These authors showed that the number of eggs laid by *B. tabaci* was always much higher on the abaxial than on the adaxial leaf surface. Also, *B. tabaci* oviposition was concentrated mostly near non-glandular trichomes, thus demonstrating that whiteflies can discriminate among the trichomes and avoid the presence of glandular trichome exudates.

Not only whiteflies but other pierce-sucking insects avoid settling on leaves in the presence of glandular trichomes. Goffreda et al. [10] suggested that the resistance of *S. pennellii* to the potato aphid is conditioned by the presence of feeding deterrent acylsugars on the leaf surface, and that these compounds appeared to be associated with the presence of type IV glandular trichomes. Furthermore, acylsugars deterred aphid settling when applied to a synthetic feeding membrane and were responsible of the modification in feeding behavior. The presence of glandular trichomes secreting alarm pheromone in the wild potato, *Solanum berthaultii* repels aphid settlement and induces rapid dispersal of settled aphid colonies [38]. A rather similar situation occurs with type I glandular trichome exudates secreted by the melon genotype TGR 1551 that deters settling of the melon aphid [39]. Similarly, we observed that type IV glandular trichomes and associated acylsucroses present on the abaxial leaf surface of ABL 14-8 tomato plants alter the settling behavior and early probing activities of *B. tabaci* when searching for an appropriate feeding site. Whitefly probing response on the abaxial surface of ABL 14-8 was characterized by an extended duration of non-probing time when compared to that on the adaxial surface. Also, the duration of probing per insect was longer on the adaxial than on the abaxial surface of ABL 14-8. All these results reflect the difficulties that *B. tabaci* has to settle and select an appropriate feeding site when exposed to ABL 14-8 leaves.

When EPG variables on adaxial and abaxial leaflet surfaces of the *B. tabaci*-susceptible cv. Moneymarker were compared, no significant differences were observed in the duration of the non-probing or probing period. However, in this genotype, whiteflies took much longer to reach the phloem from the adaxial than from the abaxial side of the leaf perhaps because of differences in the thickness of the epidermal cuticle. Thickness of the epidermal cuticle was shown to affect probing of another whitefly species on lemon leaves, for which penetration was less successful or failed when the cuticle was thick [40]. No information on differences in cuticle thickness between the adaxial and abaxial sides of tomato leaves is available and we cannot verify such hypothesis. Also, histological differences between the mesophyll of the adaxial and abaxial side of the leaves of tomato plants may account for the differences observed in the feeding behavior of *B. tabaci*. Thus, while the adaxial mesophyll has a characteristic palisade parenchyma, the abaxial mesophyll side is covered with a spongy parenchyma. Pierce-sucking insects such as whiteflies may penetrate their stylets much easier between the empty intercellular spaces of the spongy parenchyma thus reaching faster the phloem tissue from the abaxial surface of the leaves. This could result in the preference of *B. tabaci* individuals to feed from the abaxial side of the leaf in the susceptible cv. Moneymaker as they will find their target feeding site much easier.

EPG results revealed no significant differences in *B. tabaci* probing behavior on ‘Moneymaker’ and ABL 14-8 at the adaxial leaf surface probably because the major differences in epicuticular traits between the susceptible and the resistant genotypes occurred only at the abaxial leaf surface. Also, no significant differences in EPG variables were observed when whiteflies fed on the abaxial surface of both genotypes at the four-leaf growth stage (data not shown). Therefore, EPG results revealed that differences in the behavior of *B. tabaci* between ABL 14-8 and Moneymaker plants occurred only at the ten-leaf growth stage, when full expression of the acylsucrose secretion occurred.

Another set of parameters that showed significant differences between adaxial and abaxial leaf surfaces of ‘Moneymaker’ were phloem-related. *B. tabaci* individuals needed a longer time to reach the phloem on the adaxial than on the abaxial surface which could be an indirect effect of thickness of the epidermal cuticle. Thus, as observed in the interaction of aphids with tomato plants incorporating similar characters derived from *S. pennelli* [9], [10], differences in feeding preference between abaxial and adaxial surfaces occurred mainly at the epidermal level. Therefore, the differences found in the resistant genotype at the phloem level could be a consequence of the differences found in the epidermis that affects pre-phloem-related parameters such as non-probing time.

In our experiments, *B. tabaci* failed to ingest from the phloem sieve elements when exposed to the abaxial side of the ABL 14-8 leaves (Table 2) as no E2 events where registered in any of the recordings. This failure of *B. tabaci* to feed from the phloem could be related to the long time spent in non-probing activities due to the presence of acylsucroses, which deters settling and probing. As a consequence, whiteflies were unable to feed from their target tissue in the 6-hour period of EPG recording. If phloem feeding is impeded, the acquisition of a phloem-restricted virus will not occur in such time interval. Hence, the presence of glandular trichomes in ABL 14-8 plants will delay and reduce the chances of acquisition and spread of a phloem-restricted virus such as TYLCV. In a previous study, viruliferous whiteflies released on caged healthy ABL 14-8 plants were able to transmit TYLCV although less efficiently than on ‘Moneymaker’ plants [29]. Moreover, healthy whiteflies acquired the virus from infected plants, but much less efficiently on ABL 14-8 than on ‘Moneymaker’.

Furthermore, *B. tabaci* would become more vulnerable to natural enemies when avoiding the presence of acylsucroses secreted by the glandular trichomes. It is well known that the presence of trichomes or tomentose leaves hampers predator activity and natural enemies become unable to display their optimal searching activity. This has been shown for *Delphastus catalinae*, an important coccinellid predator of *B. tabaci* [41]. Any whiteflies that attempt to feed on ABL 14-8 plants will have difficulties to settle and feed on their natural and preferred feeding site, the abaxial side of leaves. When moving to the adaxial surface, whiteflies will become more exposed to the action of natural enemies that will have easier access to prey on more glabrous surfaces.

In conclusion, our results show that the behavior of *B. tabaci* is to select the abaxial side of leaves as a settling and feeding site when exposed to a susceptible genotype such as tomato line ‘Moneymarker’. However, no preference was observed on the resistant ABL 14-8 tomato plants at the ten-leaf growth stage because of the presence of glandular trichomes and their acylsucrose exudates on the abaxial side of the leaves. Such change in the innate behavior of *B. tabaci* when exposed to ABL 14-8 plants would delay and reduce the chances of transmission of a phloem-restricted virus such as TYLCV. Also, an increase in the vulnerability of *B. tabaci* to its natural enemies should be expected when exposed to ABL 14-8 plants. Additional control measures (eg. physical barriers, floating row covers, insecticides, genetic virus resistance, etc.) should be used to protect young resistant plants from *B. tabaci* and *B. tabaci*-transmitted virus diseases.
